# Supplementary figures and images for: Non-peptide dysbiosis metabolites reprogram a peptide quorum-sensing receptor to induce sustained predation in beneficial streptococci
Source: PLoS Biol. 2026 Mar 13;24(3):e3003718. doi: 10.1371/journal.pbio.3003718 (PMC12998947; doi:10.1371/journal.pbio.3003718)

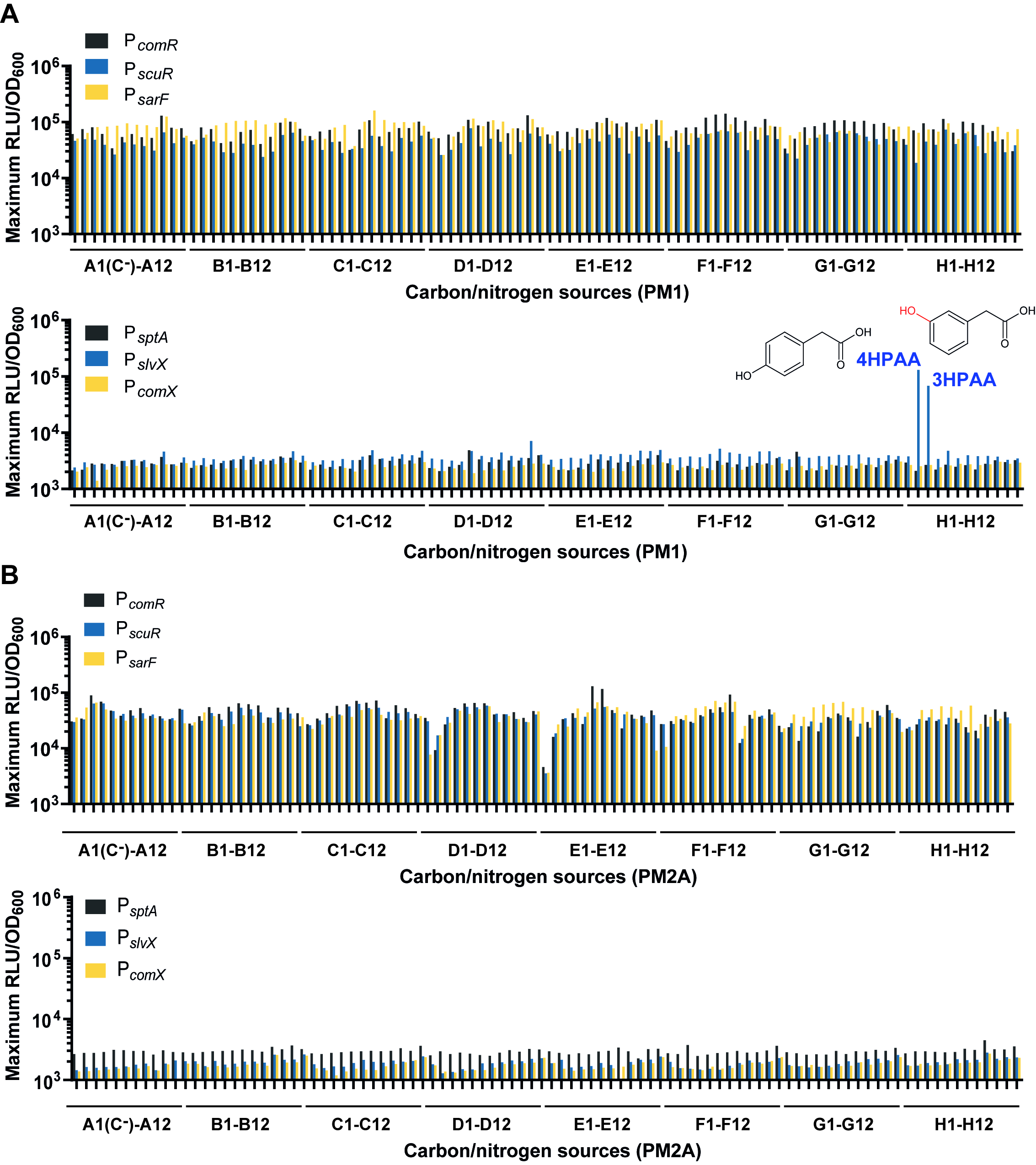

Supplement: S1 Fig — Data were obtained with Biolog plates PM1 (A) and PM2A (B). PcomR, PscuR, PsarF, PsptA, PslvX, and PcomX reporter fusions (luxAB) were tested for maximum light emission (RLU/OD600) in CDM supplemented with 0.15% glucose. Both 4-hydroxyphenylacetic acid (4HPAA) and 3-hydroxyphenylacetic acid (3HPAA) increased luminescence of PslvX, but none of the other promoters. Carbon/nitrogen sources are listed in S1 Table. The data underlying this Figure can be found in S1 Data. (TIF) [file pbio.3003718.s001.tif]

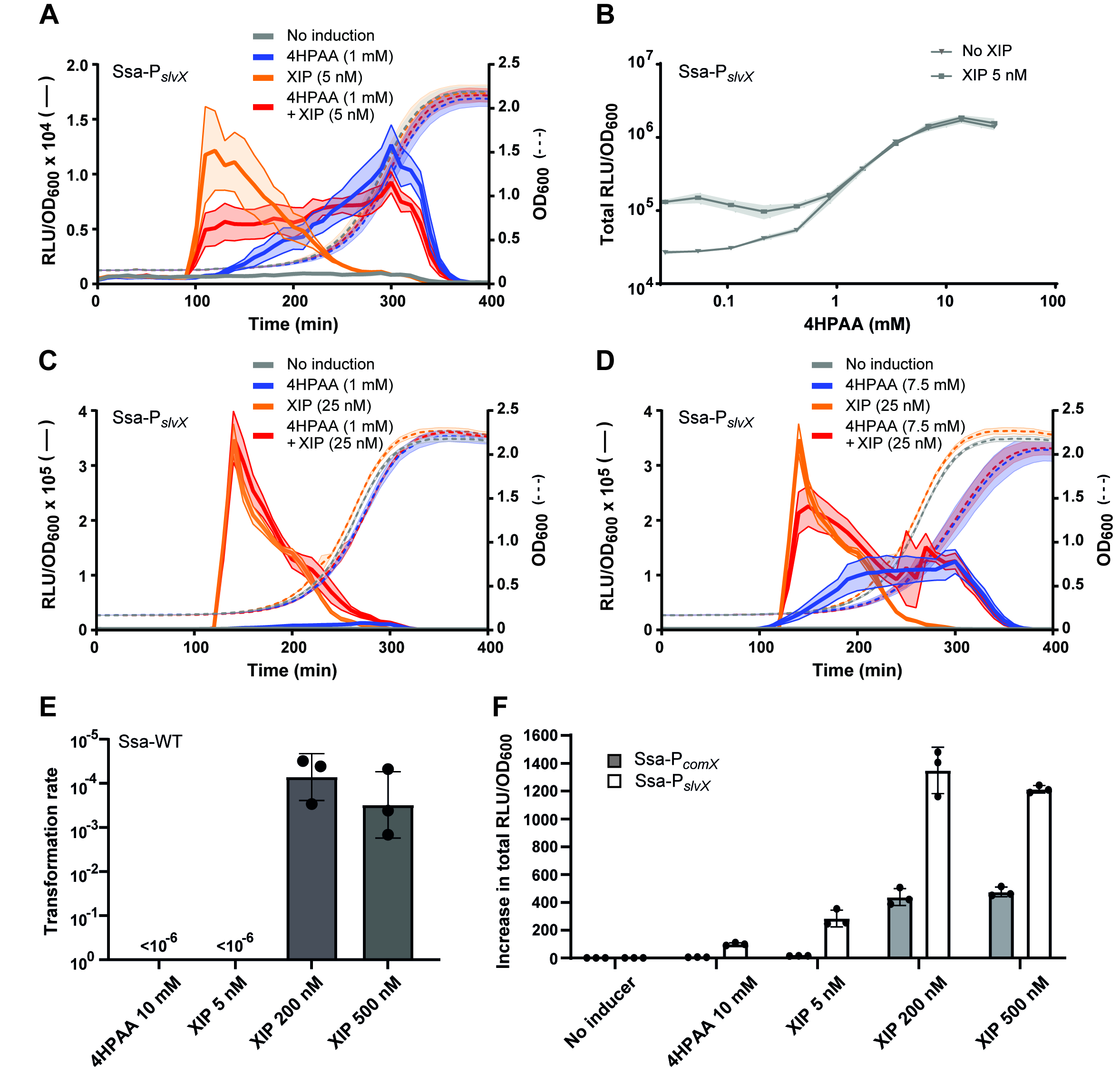

Supplement: S2 Fig — A. Luminescence (RLU/OD600) over time of the S. salivarius (Ssa) PslvX-luxAB reporter fusion without inducer, with 4HPAA (1 mM), XIP (5 nM), or 4HPAA (1 mM) + XIP (5 nM). B. Total luminescence (RLU/OD600) in response to a 4HPAA gradient (0–30 mM) in absence (No XIP) or addition of XIP (5 nM) for S. salivarius PslvX-luxAB. C. Luminescence over time of PslvX activation without inducer, with 4HPAA (1 mM), XIP (25 nM), or 4HPAA (1 mM) + XIP (25 nM). D. Luminescence over time of PslvX activation without inducer, with 4HPAA (7.5 mM), XIP (25 nM), or 4HPAA (7.5 mM) + XIP (25 nM). E. S. salivarius HSISS4 (WT) transformation assays with 4HPAA (10 mM) or XIP (5, 200, and 500 nM), reported to 1 μg of donor DNA per ml. F. Fold increase in total luminescence between 4HPAA (10 mM) or XIP (5, 200, and 500 nM) and non-induced conditions for S. salivarius PslvX or PcomX reporter strain. Data are mean values of biological triplicates ± standard deviation (light color zones in panels A-D). The data underlying this Figure can be found in S1 Data. (TIF) [file pbio.3003718.s002.tif]

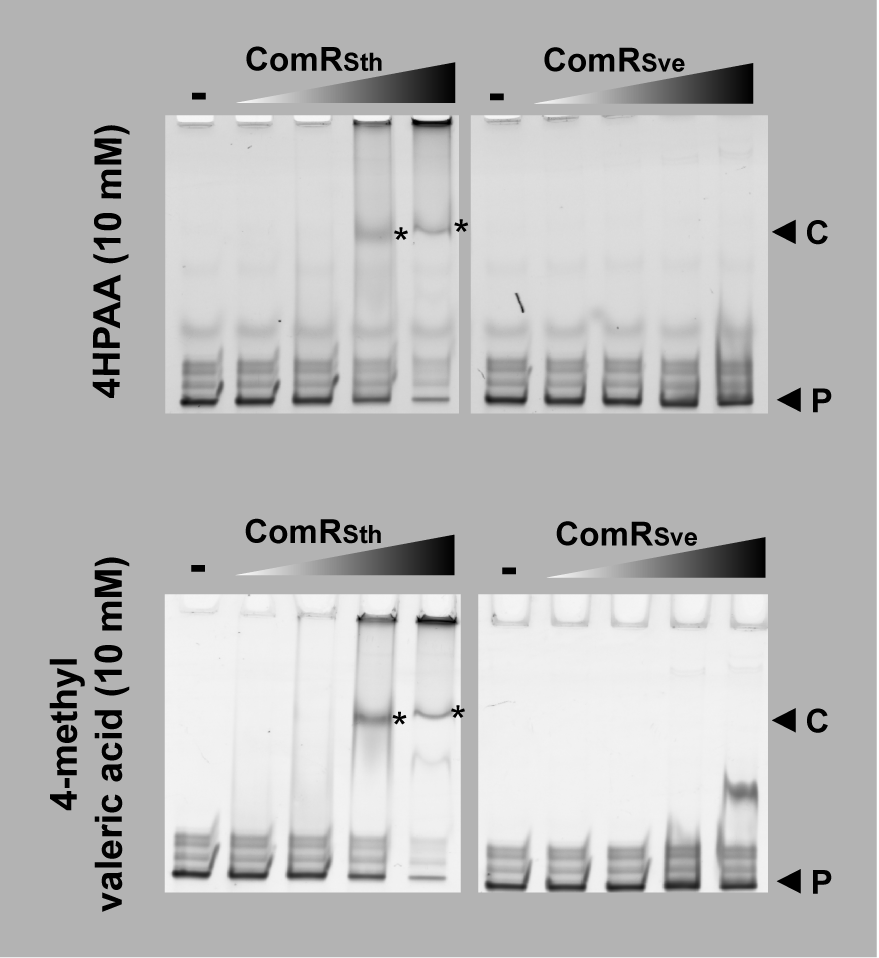

Supplement: S3 Fig — Mobility shift assays of the comS promoter probe (40 ng) conducted with gradients of purified ComRSth (left) and ComRSve (right) (Gray triangles, 2:2 dilutions from 4 μM) with 10 mM 4HPAA or 4-methylvaleric acid in the running buffer. Black arrows with a P label are the positions of the probes consisting of Cy3-conjugated DNA fragments of 40 bp. Black arrows with a C label are the position of specific complexes and stars label complexes formed with ComRSth in presence of organic acids that are not formed with ComRSve (negative control). The control condition without ComR is indicated with a minus sign (−). For each organic acid, the two parts correspond to samples that were run on the same gel. (TIF) [file pbio.3003718.s003.tif]

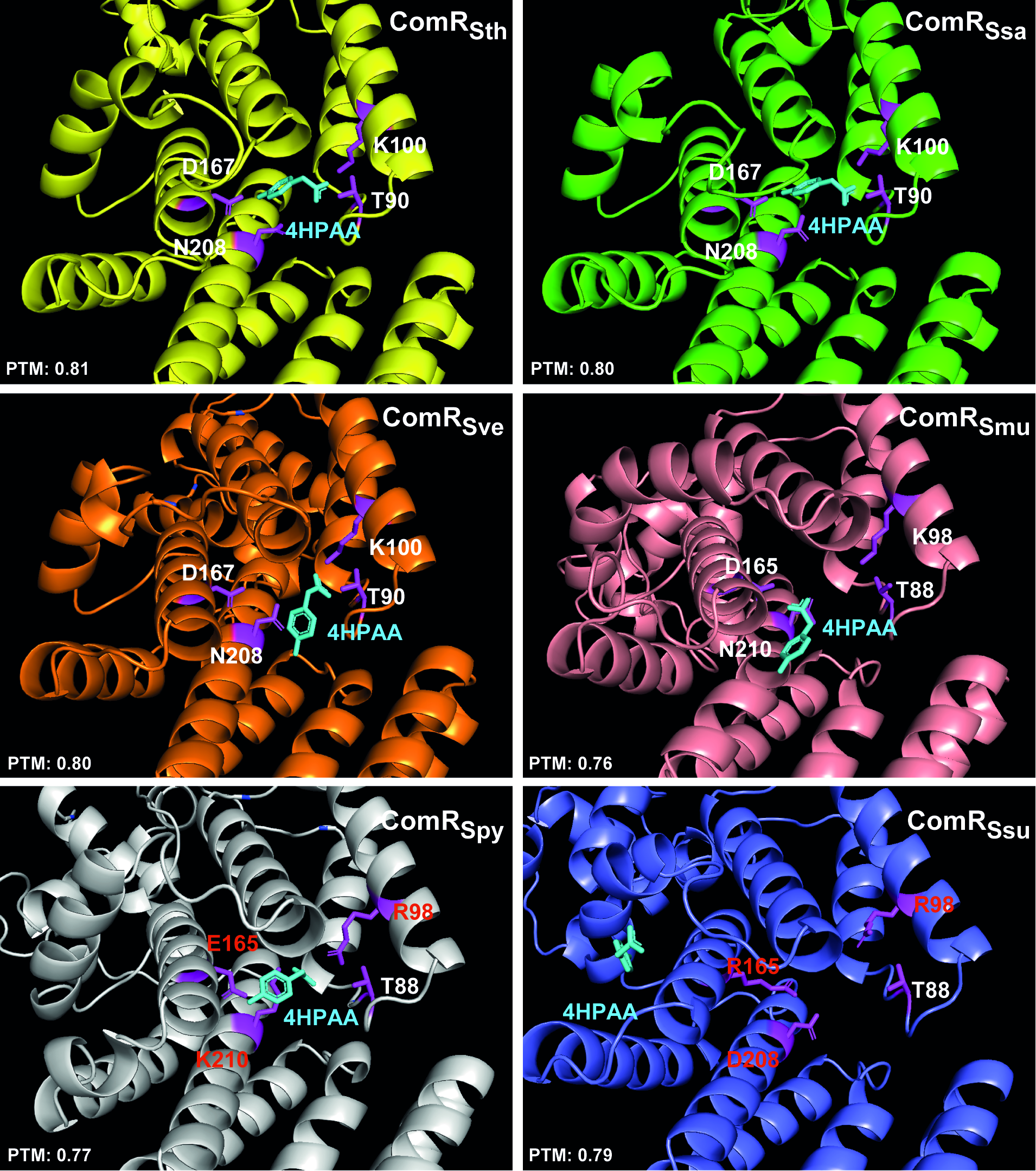

Supplement: S4 Fig — Representative ComRs are from S. salivarius HSISS4 (ComRSsa, GenBank: ALR79229.1), S. thermophilus LMD-9 (ComRSth, GenBank: ABJ65625.1), S. vestibularis F0396 (ComRSve, GenBank: EFQ60116.1), Streptococcus mutans UA159 (ComRSmu, GenBank: AAN57849.1), Streptococcus pyogenes M1 (ComRSpy, GenBank: XXA72633.1), and Streptococcus suis P1/7 (ComRSsu, GenBank: CAR44181.1). Candidate residues for interaction with 4HPAA (light blue) in ComRSth (T90, K100, D167, and N208) or at the same positions in other ComR predicted structures are higlighted in magenta. Residues labeled in red are not conserved. Modeling was performed with the protein-ligand prediction tool Chai-1. (TIF) [file pbio.3003718.s004.tif]

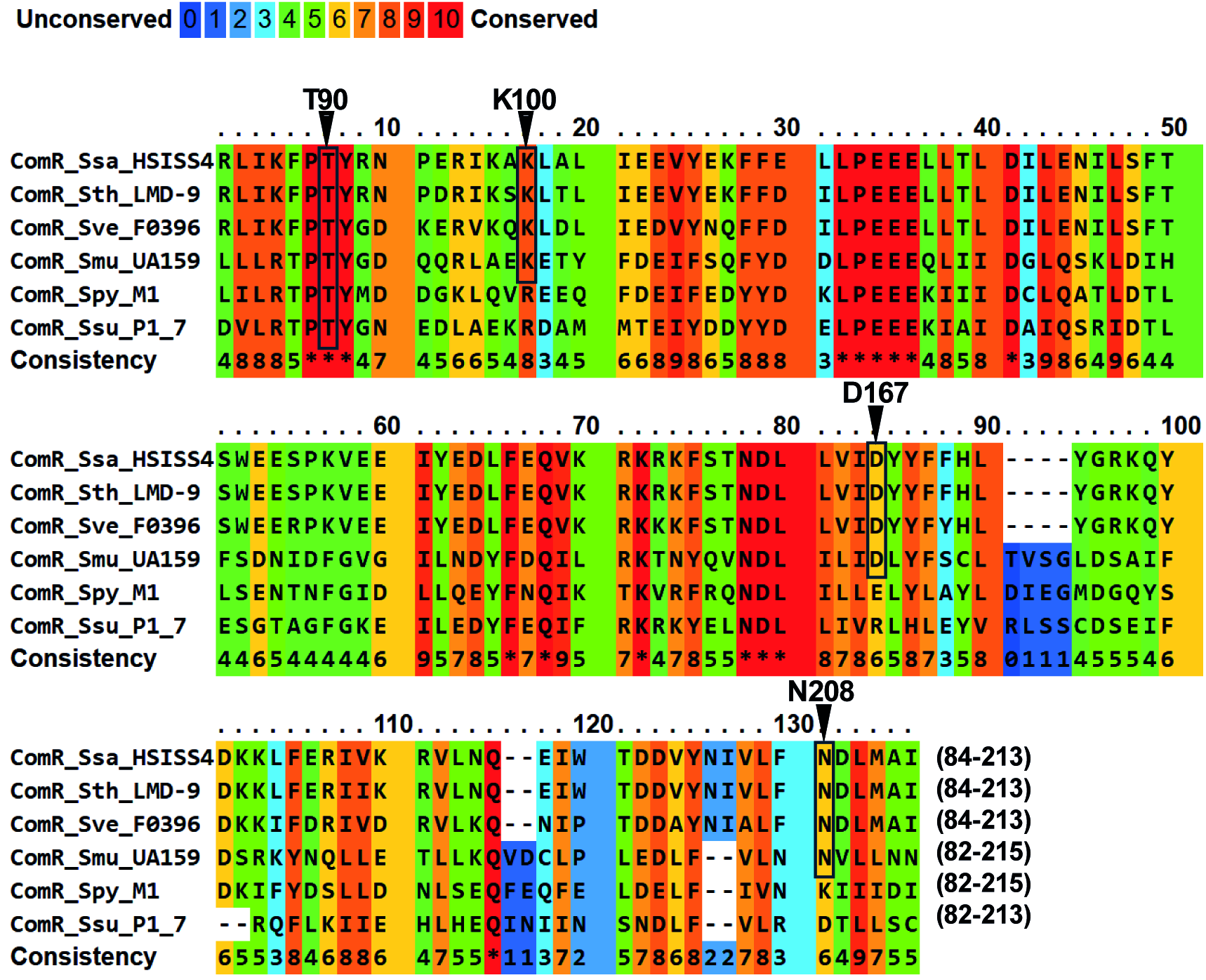

Supplement: S5 Fig — Selected ComR regions (amino-acid positions in parentheses, bottom right) are from S. salivarius HSISS4 (ComR_Ssa_HSISS4), S. thermophilus LMD-9 (ComR_Sth_LMD-9), S. vestibularis F0396 (ComR_Sve_F0396), Streptococcus mutans UA159 (ComR_Smu_UA159), Streptococcus pyogenes M1 (ComR_Spy_M1), and Streptococcus suis P1/7 (ComR_Ssu_P1_7). Candidate residues for interaction with 4HPAA in ComRSth (T90, K100, D167, and N208) are boxed. The multiple sequence alignment was performed with the PRALINE program. The color code for the conservation score (scale 1–10) is indicated on the top. (TIF) [file pbio.3003718.s005.tif]

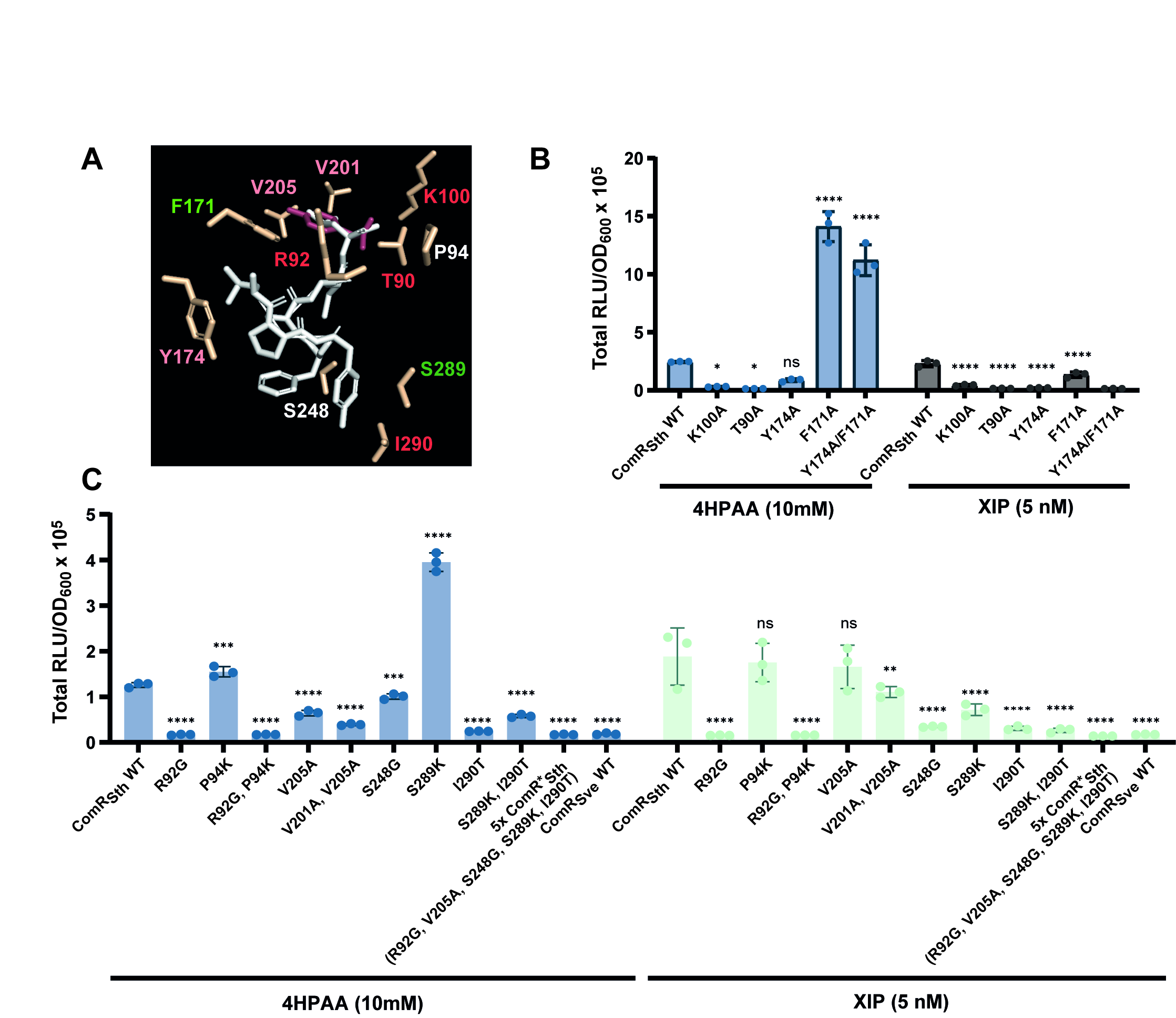

Supplement: S6 Fig — A. Positioning of the mutated ComRSth residues in the XIPSth binding pocket. Mutated residues strongly or partially affecting 4HPAA induction are labeled in red and pink, respectively. Mutated residues showing enhanced 4HPAA activation are labeled in green. Mutated residues, which are neutral regarding 4HPAA induction, are labeled in white. XIPSth and predicted 4HPAA docking are shown in white and burgundy red, respectively. B. In vivo luminescence response (total RLU/OD600) of ComRSth wild-type (WT) and variants K100A, T90A, Y174A, F171A, and Y174A/F171A to 4HPAA (10 mM) or XIP (5 nM). Experiments were performed with S. thermophilus PcomS-luxAB ΔcomS. C. In vivo luminescence response of ComRSth wild-type (WT), ComRSth penta-mutant (5× ComR*Sth), comRSve WT, and variants R92G, P94K, R92A/P94K, V205A, V201A/V205A, S248G, I290T to 4HPAA (10 mM) or XIP (5 nM). The genetic background used as in panel B. In panels B and C, dots, bars, and error bars show biological triplicates, mean values, and standard deviations, respectively. All variants were statistically compared to ComRSth WT using one-way ANOVA with Dunnett’s test (* P < 0.05; *** P < 0.001; **** P < 0.0001; ns, non-significant). The data underlying this Figure can be found in S1 Data. (TIF) [file pbio.3003718.s006.tif]

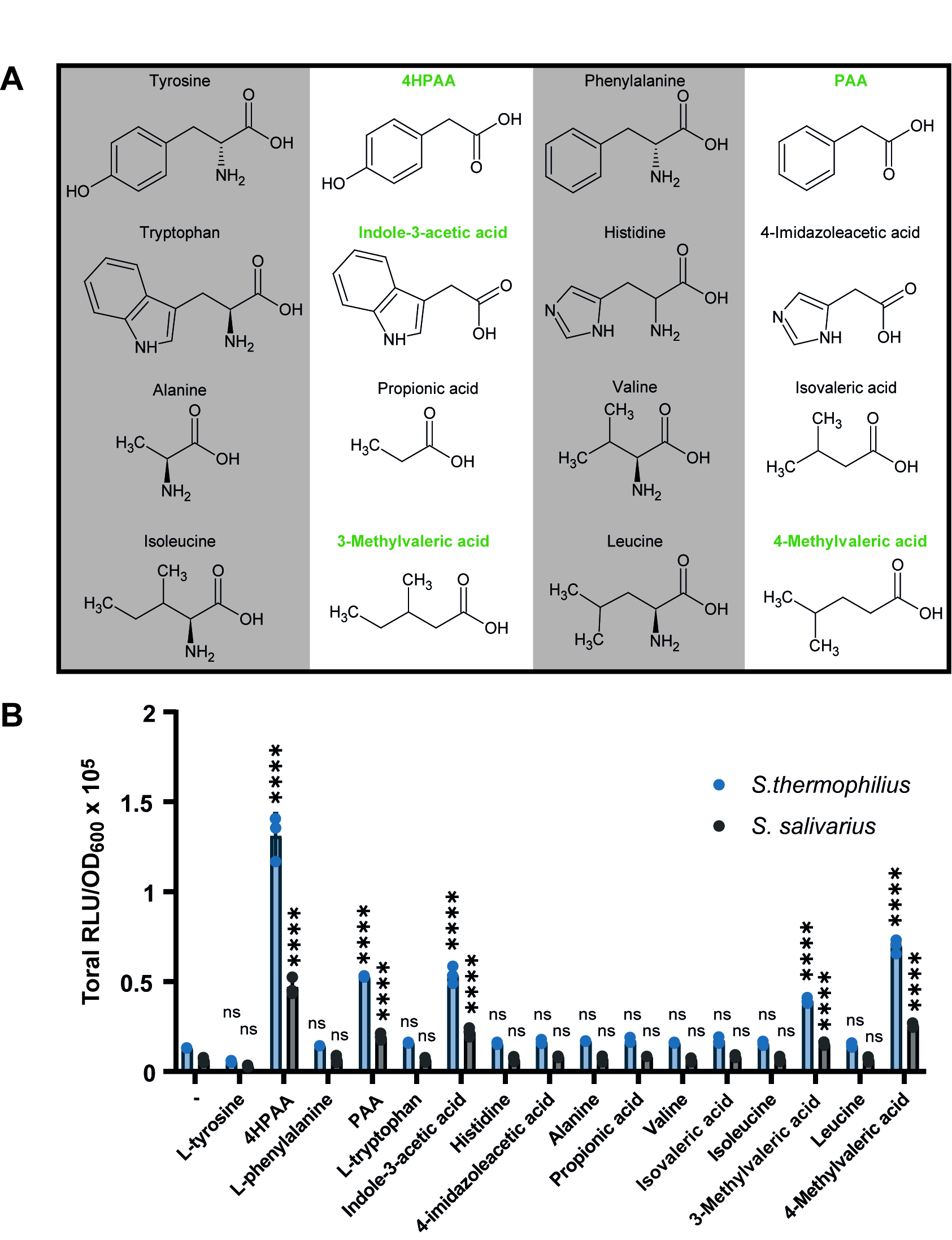

Supplement: S7 Fig — A. Structure of hydrophobic amino acids and their organic acid derivatives. The name of chemicals in bold green indicates inducing molecules. B. Luminescence assays (total RLU/OD600) with hydrophobic amino acids and their derivatives (10 mM) shown in panel A. Derivatives of Tyr, Phe, Trp, Ile, and Leu induced a signal. Experiments were performed with S. thermophilus PcomS-luxAB ΔcomS and S. salivarius PslvX-luxAB. Dots, bars, and error bars show biological triplicates, mean values, and standard deviations, respectively. All variant molecules were statistically compared to their respective control condition without addition of amino acid or organic acid derivative (minus sign) using one-way ANOVA with Dunnett’s test (**** P < 0.0001; ns, non-significant). The data underlying this Figure can be found in S1 Data. (TIF) [file pbio.3003718.s007.tif]

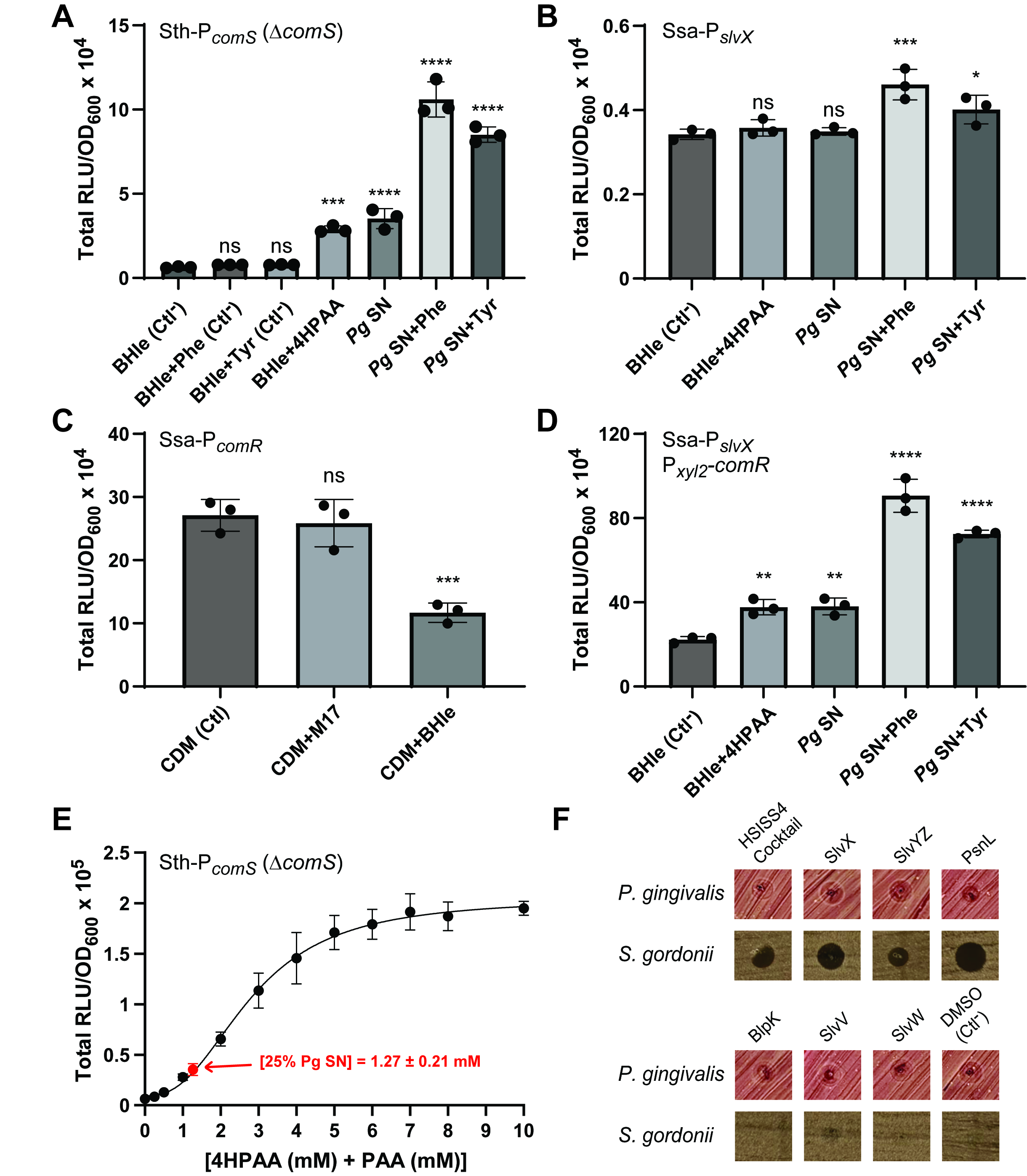

Supplement: S8 Fig — A. Luminescence assays (total RLU/OD600) of S. thermophilus (Sth) PcomS-luxAB ΔcomS with 25% (v/v) of uncultured BHIe medium without and with Phe (15 mM), Tyr (15 mM), or 4HPAA (1 mM) (negative and positive controls), filtered P. gingivalis supernatant (Pg SN), and Pg SN with Phe (15 mM) or Tyr (15 mM) in CDM. B. Luminescence assays of S. salivarius (Ssa) PslvX-luxAB as reported in panel A. C. Luminescence assays of S. salivarius PcomR-luxAB without (CDM, Ctl) and with 25% (v/v) M17 or BHIe. D. Luminescence assays of S. salivarius PslvX-luxAB Pxyl2-comR as reported in panel B. The comR expression was induced by adding 0.2% xylose. E. Luminescence assays of S. thermophilus PcomS-luxAB ΔcomS with increasing concentration (mM) of a mixture of 4HPAA and PAA (ratio 1:1). Each compound of the mixture was individually resuspended in BHIe medium at the indicated concentration. Twenty-five% (v/v) of BHIe solutions were incorporated in CDM. The red arrow corresponds to the concentration detected in Pg SN. F. Bacteriocin assays with synthetic versions of salivaricins from S. salivarius HSISS4 either used either as a cocktail of 6 peptides or tested separately. The broad-spectrum salivaricin PsnL (not produced by HSISS4) was also tested. P. gingivalis W83 and S. gordonii LMG 17,843 were used as indicator strains. DMSO (100%) was used as negative control. In panels A to D, dots, bars, and error bars show biological triplicates, mean values, and standard deviations, respectively. All tested conditions were statistically compared to the control (Ctl) using a one-way ANOVA with Dunnett’s test (* P < 0.05; ** P < 0.01; *** P < 0.001; **** P < 0.0001; ns, non-significant). The data underlying this Figure can be found in S1 Data. (TIF) [file pbio.3003718.s008.tif]

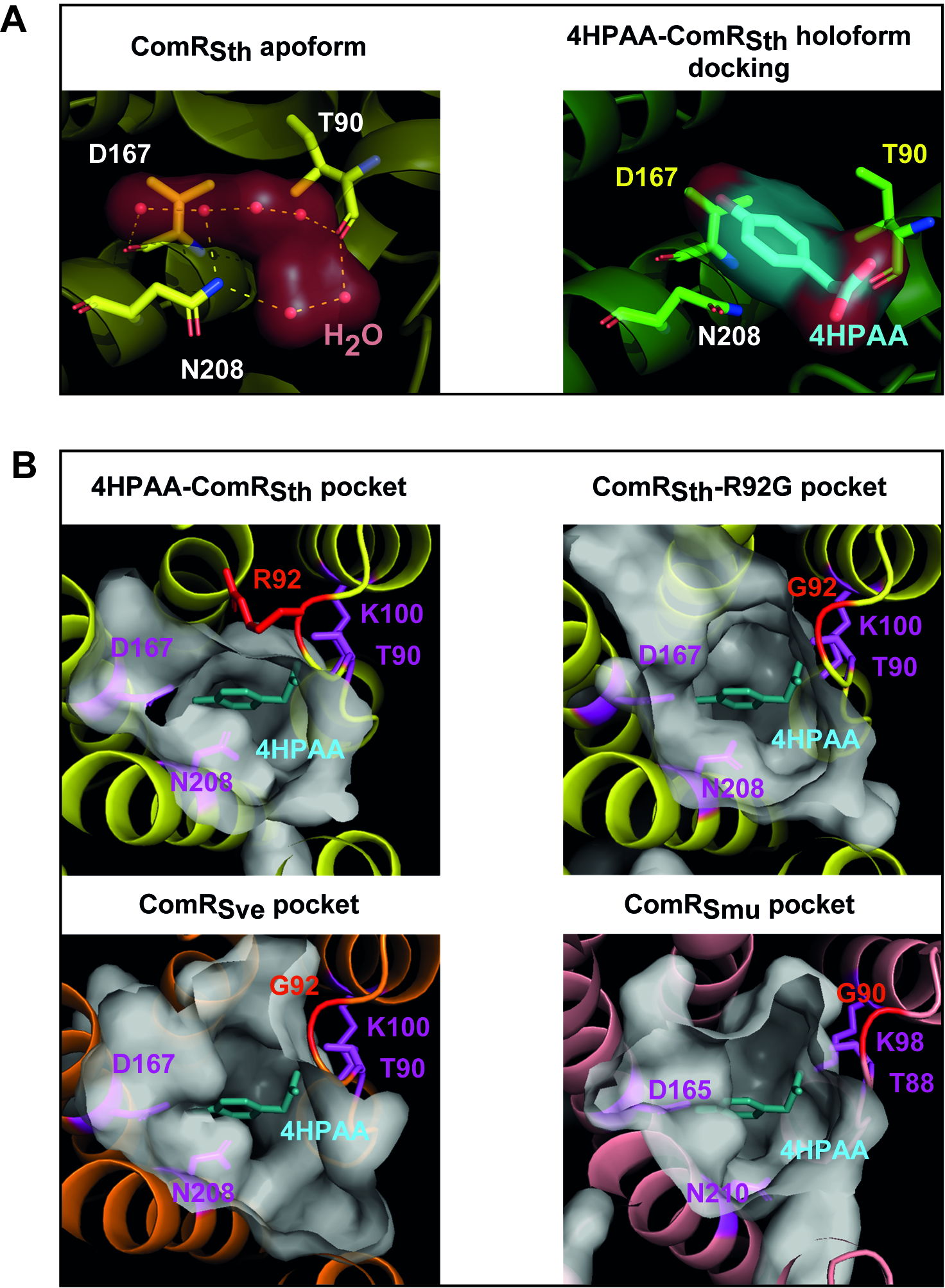

Supplement: S9 Fig — A. Position of water molecules (red spheres) in the bottom of the XIP-binding pocket of ComRSth apoform (PDB 5JUF) (left) compared to the predicted docking of 4HPAA (blue) in ComRSth holoform (PDB 5JUB) (right). Four out of six water molecules are interacting with residues T90, D167, and N208 in the apoform (in yellow), which are also predicted to interact with 4HPAA in the holoform (in green). B. Comparison of the size of the 4HPAA-binding pocket (light gray) between holo-ComRSth (PDB 5JUB), ComRSth-R92G (AlphaFold2 model), holo-ComRSve (PDB 6HUA), and ComRSmu (AlphaFold2 model). The R92G substitution, which abolished 4HPAA induction in ComRSth, is highlighted in red. Residues T90, K100, D167, and N208 (or their equivalent positions) interacting with 4HPAA are colored in mauve. The position of docked 4HPAA (blue-green) in holo-ComRSth is reported in the other structures. (TIF) [file pbio.3003718.s009.tif]
