# Supplementary material for: Non-peptide dysbiosis metabolites reprogram a peptide quorum-sensing receptor to induce sustained predation in beneficial streptococci
Source: PLoS Biol. 2026 Mar 13;24(3):e3003718. doi: 10.1371/journal.pbio.3003718 (PMC12998947; doi:10.1371/journal.pbio.3003718)
Supplement: S1 Table — (PDF) [file pbio.3003718.s010.pdf]

S1 Table. List of tested carbon/nitrogen sources from Biolog plates PM1 and PM2A.

Plate #	Carbon/nitrogen source	Plate #	Carbon/nitrogen source
**Biolog plate PM1**		**Biolog plate PM2A**	
A1	- (control)	A1	- (control)
A2	L-Arabinose	A2	Chondroitin-Chondroitin Sulfate C
A3	N-Acetyl-D-Glucosamine	A3	α-Cyclodextrin
A4	D-Saccharic Acid	A4	ß-Cyclodextrin
A5	Succinic Acid	A5	γ-Cyclodextrin
A6	D-Galactose	A6	Dextrin
A7	L-Aspartic Acid	A7	Gelatin
A8	L-Proline	A8	Glycogen
A9	D-Alanine	A9	Inulin
A10	D-Trehalose	A10	Laminarin
A11	D-Mannose	A11	Mannan
A12	Dulcitol	A12	Pectin
B1	D-Serine	B1	N-Acetyl-D-Galactosamine
B2	D-Sorbitol	B2	N-Acetyl-Neuraminic Acid
B3	Glycerol	B3	ß-D-Allose
B4	L-Fucose	B4	Amygdalin
B5	D-Glucuronic Acid	B5	D-Arabinose
B6	D-Gluconic Acid	B6	D-Arabitol
B7	D,L-α-Glycerol- Phosphate	B7	L-Arabitol
B8	D-Xylose	B8	Arbutin
B9	L-Lactic Acid	B9	2-Deoxy-DRibose
B10	Formic Acid	B10	i-Erythritol
B11	D-Mannitol	B11	D-Fucose
B12	L-Glutamic Acid	B12	3-0-ß-DGalactopyranosylD-Arabinose
C1	D-Glucose-6-Phosphate	C1	Gentiobiose
C2	D-Galactonic Acid-γ-Lactone	C2	L-Glucose
C3	D,L-Malic Acid	C3	Lactitol
C4	D-Ribose	C4	D-Melezitose
C5	Tween 20	C5	Maltitol
C6	L-Rhamnose	C6	α-Methyl-DGlucoside
C7	D-Fructose	C7	ß-Methyl-DGalactoside
C8	Acetic Acid	C8	3-Methyl Glucose
C9	α-D-Glucose	C9	ß-Methyl-DGlucuronic Acid
C10	Maltose	C10	α-Methyl-DMannoside
C11	D-Melibiose	C11	ß-Methyl-DXyloside
C12	Thymidine	C12	Palatinose
D1	L-Asparagine	D1	D-Raffinose
D2	D-Aspartic Acid	D2	Salicin
D3	D-Glucosaminic Acid	D3	Sedoheptulosan
D4	1,2-Propanediol	D4	L-Sorbose
D5	Tween 40	D5	Stachyose
D6	α-Keto-Glutaric Acid	D6	D-Tagatose
D7	α-Keto-Butyric Acid	D7	Turanose
D8	α-Methyl-DGalactoside	D8	Xylitol

D9	α-D-Lactose
D10	Lactulose
D11	Sucrose
D12	Uridine
E1	L-Glutamine
E2	m-Tartaric Acid
E3	D-Glucose-1- Phosphate
E4	D-Fructose-6- Phosphate
E5	Tween 80
E6	α-Hydroxy Glutaric Acid-γLactone
E7	α-Hydroxy Butyric Acid
E8	ß-Methyl-DGlucoside
E9	Adonitol
E10	Maltotriose
E11	2-Deoxy Adenosine
E12	Adenosine
F1	Glycyl-L-Aspartic Acid
F2	Citric Acid
F3	myo-Inositol
F4	D-Threonine
F5	Fumaric Acid
F6	Bromo Succinic Acid
F7	Propionic Acid
F8	Mucic Acid
F9	Glycolic Acid
F10	Glyoxylic Acid
F11	D-Cellobiose
F12	Inosine
G1	Glycyl-LGlutamic Acid
G2	Tricarballylic Acid
G3	L-Serine
G4	L-Threonine
G5	L-Alanine
G6	L-Alanyl-Glycine
G7	Acetoacetic Acid
G8	N-Acetyl-ß-DMannosamine
G9	Mono Methyl Succinate
G10	Methyl Pyruvate
G11	D-Malic Acid
G12	L-Malic Acid
H1	Glycyl-L-Proline
H2	**p-Hydroxy Phenyl Acetic Acid**
H3	**m-Hydroxy Phenyl Acetic Acid**
H4	Tyramine
H5	D-Psicose
H6	L-Lyxose
H7	Glucuronamide
H8	Pyruvic Acid
H9	L-Galactonic Acid-γ-Lactone

D9	N-Acetyl-DGlucosaminitol
D10	γ-Amino Butyric Acid
D11	δ-Amino Valeric Acid
D12	Butyric Acid
E1	Capric Acid
E2	Caproic Acid
E3	Citraconic Acid
E4	Citramalic Acid
E5	D-Glucosamine
E6	2-Hydroxy Benzoic Acid
E7	4-Hydroxy Benzoic Acid
E8	ß-Hydroxy Butyric Acid
E9	Glycolic Acid
E10	α-Keto-Valeric Acid
E11	Itaconic Acid
E12	5-Keto-DGluconic Acid
F1	D-Lactic Acid Methyl Ester
F2	Malonic Acid
F3	Melibionic Acid
F4	Oxalic Acid
F5	Oxalomalic Acid
F6	Quinic Acid
F7	D-Ribono-1,4- Lactone
F8	Sebacic Acid
F9	Sorbic Acid
F10	Succinamic Acid
F11	D-Tartaric Acid
F12	L-Tartaric Acid
G1	Acetamide
G2	L-Alaninamide
G3	N-Acetyl-LGlutamic Acid
G4	L-Arginine
G5	Glycine
G6	L-Histidine
G7	L-Homoserine
G8	Hydroxy-LProline
G9	L-Isoleucine
G10	L-Leucine
G11	L-Lysine
G12	L-Methionine
H1	L-Ornithine
H2	L-Phenylalanine
H3	L-Pyroglutamic Acid
H4	L-Valine
H5	D,L-Carnitine
H6	Sec-Butylamine
H7	D,L-Octopamine
H8	Putrescine
H9	Dihydroxy Acetone

H10	D-Galacturonic Acid	H10	2,3-Butanediol
H11	Phenylethylamine	H11	2,3-Butanedione
H12	2-Aminoethanol	H12	3-Hydroxy-2- Butanone
