# Supplementary material for: Non-peptide dysbiosis metabolites reprogram a peptide quorum-sensing receptor to induce sustained predation in beneficial streptococci
Source: PLoS Biol. 2026 Mar 13;24(3):e3003718. doi: 10.1371/journal.pbio.3003718 (PMC12998947; doi:10.1371/journal.pbio.3003718)
Supplement: S2 Table — (PDF) [file pbio.3003718.s011.pdf]

S2 Table. List of organic acids and amino acids tested in this study.

Common name	Systematic IUPAC	Abbreviation	Supplier
4-hydroxy phenylacetic acid	(4-Hydroxyphenyl)acetic acid	4HPAA	Thermo Fisher Scientific
3-Hydroxy phenylacetic acid	2-(3-Hydroxyphenyl)acetic acid	3HPAA	Merck
Tyramine	4-(2-Aminoethyl)phenol		Thermo Fisher Scientific
Methyl 4-hydroxyphenylacetate	Methyl 2-(4-hydroxyphenyl)acetate	Methyl-4HPAA	Thermo Fisher Scientific
D-(+)-3-Phenyllactic acid	(R)-2-Hydroxy-3-phenylpropionic acid		Thermo Fisher Scientific
(R)-(-)-Mandelic Acid	(2R)-2-Hydroxy-2-phenylacetic acid		Thermo Fisher Scientific
Phenylacetic acid	2-Phenylethanoic acid	PAA	Merck
3-Fluoro-4-hydroxyphenylacetic acid	2-(3-Fluoro-4-hydroxyphenyl)acetic acid	3-Fluoro-4HPAA	Thermo Fisher Scientific
Pentafluoro phenylacetic acid	2,3,4,5,6-Pentafluorophenylacetic acid	Pentafluoro-PAA	Thermo Fisher Scientific
Homovanillic acid	(4-Hydroxy-3-methoxyphenyl)acetic acid		Thermo Fisher Scientific
4-Hydroxy-3,5-dimethoxyphenylacetic acid	2-(4-Hydroxy-3,5-dimethoxyphenyl)acetic acid	4-Hydroxy-3,5-dimethoxy-PAA	Thermo Fisher Scientific
1-Naphthylacetic acid	2-(Naphthalen-1-yl)acetic acid		Thermo Fisher Scientific
Indole-3-acetic acid	2-(1H-indol-3-yl)acetic acid	IAA	Thermo Fisher Scientific
3-Methylvaleric acid	3-Methylpentanoic acid	3-MVA	Thermo Fisher Scientific
4-Methylvaleric acid	4-Methylpentanoic acid	4-MVA	Thermo Fisher Scientific
Isovaleric acid	3-Methylbutanoic acid		Thermo Fisher Scientific
4-Imidazolacetic acid	Butyl 2-(1*H*-imidazol-5-yl)acetate		Thermo Fisher Scientific
Propionic acid	Propionic acid		Thermo Fisher Scientific
Tyrosine	L-2-Amino-3-(4-hydroxyphenyl)propanoic acid		Thermo Fisher Scientific
Phenylalanine	(*S*)-2-Amino-3-phenylpropanoic acid		Thermo Fisher Scientific
Tryptophan	(2*S*)-2-Amino-3-(1*H*-indol-3-yl)propanoic aci		Thermo Fisher Scientific
Histidine	2-Amino-3-(1*H*-imidazol-4-yl)propanoic acid		Thermo Fisher Scientific
Alanine	2-Aminopropanoic acid		Thermo Fisher Scientific
Valine	2-Amino-3-methylbutanoic acid		Thermo Fisher Scientific
Isoleucine	(2S,3S)-2-Amino-3-methylpentanoic acid		Thermo Fisher Scientific
Leucine	2-Amino-4-methylpentanoic acid		Thermo Fisher Scientific
