# Supplementary material for: Non-peptide dysbiosis metabolites reprogram a peptide quorum-sensing receptor to induce sustained predation in beneficial streptococci
Source: PLoS Biol. 2026 Mar 13;24(3):e3003718. doi: 10.1371/journal.pbio.3003718 (PMC12998947; doi:10.1371/journal.pbio.3003718)
Supplement: S3 Table — (PDF) [file pbio.3003718.s012.pdf]

S3 Table. List of strains and plasmids.

Strain/plasmid	Genotype/description	Resistance[a]	Source
**Strains**			
Escherichia coli			
E. coli TOP10	F$^-$ *mcrA* Δ(*mrr-hsdRMS mcrBC*) φ80*lacZ*Δ*M15* Δ*lacX74* *recA1 araD139* Δ(*ara-leu*) 7697 *galU galK rpsL* (Str^r) *endA1 nupG λ-*	-	Invitrogen, CA
Streptococcus thermophilus			
LMD-9	Wild-type milk isolate	-	ATCC[b]
LF121	LMD-9 (*blpD-blpX*)::P$_{comS}$-*luxAB*	-	(1)
LF134	LF121 *ΔcomS*::P$_{32}$-*cat*	Cm^R	(1)
LF135	LF121 *ΔcomR::*P$_{32}$-*cat*	Cm^R	(1)
LF138	LF121 *ΔamiA1-amiF::*P$_{32}$-*cat*	Cm^R	L. Fontaine, Lab. collection
LF149	LF121 *comR::comR*$_{T90A}$*, ΔcomS::*P$_{32}$-*cat*	Cm^R	(2)
LF152	LF121 *comR::comR*$_{K100A}$*, ΔcomS::*P$_{32}$-*cat*	Cm^R	(2)
LF153	LF121 *comR::comR*$_{F171A-Y174A}$, *ΔcomS*::P$_{32}$-*cat*	Cm^R	(2)
LL1	LF121 *comR::comR*$_{Sve}$*, ΔcomS::P*$_{32}$-*cat*	Cm^R	(3)
LL10	LF121 *comR::comR*$_{R92G-P94K}$*, ΔcomS::*P$_{32}$-*cat; L6**	Cm^R	(3)
LL11	LF121 *comR::comR*$_{V201A-V205A}$*, ΔcomS::*P$_{32}$-*cat; α12**	Cm^R	(3)
LL12	LF121 *comR::comR*$_{S248G}$*, ΔcomS::*P$_{32}$-*cat; α14**	Cm^R	(3)
LL13	LF121 *comR::comR*$_{S289K-I290T}$*, ΔcomS::*P$_{32}$-*cat; CAP**	Cm^R	(3)
LL15	LF121 *comR::comR*$_{V205A}$*, ΔcomS::*P$_{32}$-*cat*	Cm^R	(3)
LL19	LF121 *comR::comR*$_{R92G-V205A-S248G-S289K-I290T}$*, ΔcomS::*P$_{32}$-*cat*	Cm^R	(3)
LL40	LF121 *comR::comR*$_{F171A}$*, ΔcomS::*P$_{32}$-*cat*	Cm^R	(4)
LL42	LF121 *comR::comR*$_{Y174A}$, Δ*comS*::P$_{32}$-*cat*	Cm^R	(4)
LL100	LF121 *comR::comR*$_{R92G}$*, ΔcomS::*P$_{32}$-*cat*	Cm^R	(3)
LL101	LF121 *comR::comR*$_{P94K}$*, ΔcomS::*P$_{32}$-*cat*	Cm^R	(3)
LL102	LF121 *comR::comR*$_{S289K}$*, ΔcomS::*P$_{32}$-*cat*	Cm^R	(3)
LL103	LF121 *comR::comR*$_{I290T}$*, ΔcomS::*P$_{32}$-*cat*	Cm^R	(3)
Streptococcus salivarius			
HSISS4	Wild-type gastro-intestinal tract isolate	-	(5)
JM1001	HSISS4 Δ*comR*	-	(6)
JM1013	HSISS4 Δ*slv5*	-	(6)
JM1016	HSISS4 *tRNA*^{ser}*::*P$_{xyl2}$-*comR-spc*	Spec^R	(6)
JM1020	HSISS4 *tRNA*^{Thr}*::*P$_{comX}$-*luxAB-cat*	Cm^R	(6)
JM1027	HSISS4 *tRNA*^{Thr}*::*P$_{slvX}$-*luxAB-cat*	Cm^R	(6)
JM1100	HSISS4 *tRNA*^{Thr}*::*P$_{sptA}$-*luxAB-cat*	Cm^R	(7)
JM1101	HSISS4 *tRNA*^{Ser}*::*P$_{32}$-*scuR-spc* (*scuR*$^{++}$)	Spec^R	(7)

JM1118	HSISS4 Δ*scuR-sarF::erm*	Ery^R	(7)
JM1192	JM1027 Δ*scuR-sarF::erm*	Cm^R & Ery^R	(7)
JM1175	JM1100 Δ*scuR::erm*	Ery^R	(7)
JM1300	JM1027 Δ*comS::erm*	Cm^R & Ery^R	This work
JM1301	JM1027 Δ*comR::erm*	Cm^R & Ery^R	This work
JM1302	HSISS4 $tRNA^{Thr}$::P_{comR}-*luxAB-cat*	Cm^R	This work
JM1303	HSISS4 $tRNA^{Thr}$::P_{scuR}-*luxAB-cat*	Cm^R	This work
JM1304	HSISS4 $tRNA^{Thr}$::P_{sarF}-*luxAB-cat*	Cm^R	This work
JM1305	JM1027 $tRNA^{Ser}$::P_{xyl2}-*comR-cat*	Cm^R	This work
Lactococcus lactis			
IL1403	Laboratory strain	-	(8)
Porphyromonas gingivalis			
W83 (ATCC BAA-308)	Wild-type clinical isolate	-	ATCC[b]
Streptococcus gordonii			
LMG 17843	Wild-type isolate from human oral cavity		BCCM[c]
**Plasmids**			
pBAD-$comR_{Sth}$-*strep*	pBADHisA derivative containing the translation fusion P_{ara}BAD-$comR_{Sth}$-*strep*	Ap^R	(1)
pBAD-$comR_{Sve}$-*strep*	pBADHisA derivative containing the translation fusion P_{ara}BAD-$comR_{Sve}$-*strep*	Ap^R	(3)
pGIUD0855*ery*	pUC18 derivative containing the *erm* gene	Ery^R	(9)
pJIM*cat*	pJIM4900 derivative with a *cat* cassette	Cm^R	(7)

[a]Ap^R, Cm^R, Ery^R, and $Spec^R$; resistance to ampicillin, chloramphenicol, erythromycin, and spectinomycin, respectively.
[b]ATCC, American Type Culture Collection, Rockville, MD.
[c]BCCM, Belgian Co-ordinated Collections of Micro-organisms

REFERENCES

1. Fontaine, L., Goffin, P., Dubout, H., Delplace, B., Baulard, A., Lecat-Guillet, N. *et al.* (2013) Mechanism of competence activation by the ComRS signalling system in streptococci *Mol Microbiol* **87**, 1113-1132
2. Talagas, A., Fontaine, L., Ledesma-Garcia, L., Mignolet, J., Li de la Sierra-Gallay, I., Lazar, N. *et al.* (2016) Structural Insights into Streptococcal Competence Regulation by the Cell-to-Cell Communication System ComRS *PLoS Pathog* **12**, e1005980
3. Ledesma-Garcia, L., Thuillier, J., Guzman-Espinola, A., Ensinck, I., Li de la Sierra-Gallay, I., Lazar, N. *et al.* (2020) Molecular dissection of pheromone selectivity in the competence signaling system ComRS of streptococci *Proc Natl Acad Sci U S A* **117**, 7745-7754
4. Ledesma-Garcia, L., Ensinck, I., Dereinne, D., Viela, F., Mignolet, J., Dufrene, Y. F. *et al.* (2021) Coevolution of the bacterial pheromone ComS and sensor ComR fine-tunes natural transformation in streptococci *J Biol Chem* **297**, 101346
5. Mignolet, J., Fontaine, L., Kleerebezem, M., and Hols, P. (2016) Complete Genome Sequence of *Streptococcus salivarius* HSISS4, a Human Commensal Bacterium Highly Prevalent in the Digestive Tract *Genome Announc* **4**,
6. Mignolet, J., Fontaine, L., Sass, A., Nannan, C., Mahillon, J., Coenye, T. *et al.* (2018) Circuitry Rewiring Directly Couples Competence to Predation in the Gut Dweller *Streptococcus salivarius Cell Rep* **22**, 1627-1638
7. Mignolet, J., Cerckel, G., Damoczi, J., Ledesma-Garcia, L., Sass, A., Coenye, T. *et al.* (2019) Subtle selectivity in a pheromone sensor triumvirate desynchronizes competence and predation in a human gut commensal *Elife* **8**,
8. Chopin, A., Chopin, M. C., Moillo-Batt, A., and Langella, P. (1984) Two plasmid-determined restriction and modification systems in *Streptococcus lactis Plasmid* **11**, 260-263
9. Fontaine, L., Boutry, C., de Frahan, M. H., Delplace, B., Fremaux, C., Horvath, P. *et al.* (2010) A novel pheromone quorum-sensing system controls the development of natural competence in Streptococcus thermophilus and Streptococcus salivarius *J Bacteriol* **192**, 1444-1454
