# Supplementary material for: Non-peptide dysbiosis metabolites reprogram a peptide quorum-sensing receptor to induce sustained predation in beneficial streptococci
Source: PLoS Biol. 2026 Mar 13;24(3):e3003718. doi: 10.1371/journal.pbio.3003718 (PMC12998947; doi:10.1371/journal.pbio.3003718)
Supplement: S4 Table — (PDF) [file pbio.3003718.s013.pdf]

**S4 Table. List of oligonucleotides used in this study.**

**Construction of *S. salivarius* strains**

| Names | Sequence (5' to 3') |
|---|---|
| Uplox66 | TAAGGAAGATAAATCCCATAAGG |
| DNlox71 | TTCACGTTACTAAAGGGAATGTA |
| lox66-ery | TAAGGAAGATAAATCCCATAAGGTACCTAATAATTTATCTACATTCC |
| lox71-ery | TTCACGTTACTAAAGGGAATGTAAAATGATACACCAATCAGTGC |
| UF_tRNAthr | TGTCAAAGGATTAGGAAAAC |
| UR_tRNAthr | TTGATTTATACCTCTCAATTT |
| DF_tRNAthr | AAATCAACCTCTTTGAACATA |
| DR_tRNAthr | AAAAAAGAATTCATTCATGATGAGCGGGTTCGTGAGA |
| F_luxAB_ATG | ATGAAATTTGGAAACTTTTTGC |
| R_cat_tRNAthr | TATGTTCAAAGAGGTTGATTTCACGTTACTAAAGGGAATGTA |
| UFcomRJIM-SS1-4 | GCAGTACCACTCTATGCTAAATTTGCCAACTTTGA |
| URcomRJIM-SS1-4 | CCTTATGGGATTTATCTTCCTTAGAGACACTCCTTTATTTC |
| DFcomRJIM-SS1-4 | TACATTCCCTTTAGTAACGTGAAAAATGGTGGTGACATAAA |
| DRcomRJIM-SS1-4 | TGACGTGATTTCACCAGTACGACGTGAACTAAAGA |
| Up_comR SS1-4 | TTGCTTACAGTTGCTATGGT |
| Down_comR_SS1-4 | TCATCACAATGGTCACATCT |
| UF_PcomR_luxAB | TAATTGAGGAGGTCTATGAG |
| UR_comS | CCTTATGGGATTTATCTTCCTTATAAAACTCCTTTTAACTGTTAG |
| DF_comS | TACATTCCCTTTAGTAACGTGAATAATAAGGAGTCACCATGTC |
| F_comR | CTAGAGGAGGAATTTAGATGAACATAAAAGACAGCATTG |
| Down_PcomS_JIMSS1-4 | GACAAAGTAGTCAAGACCGT |
| F_PrggD_tRNAthr | AAATTGAGAGGTATAAATCAATGCTATAATTTTCATCATCG |
| F_PrggC_tRNAthr | AAATTGAGAGGTATAAATCAATCCTATTTATAACACTGACC |
| R_PcomR_luxAB_ATG | GCAAAAAGTTTCCAAATTTCAAGAGACACTCCTTTATTT |
| R_PrggD_luxAB_ATG | GCAAAAAGTTTCCAAATTTCATACATAATTCCTTATGATTT |
| R_PrggC_luxAB_ATG | GCAAAAAGTTTCCAAATTTCATCTGTATTTCCCTTGAG |

**Transformation assays**

| Name | Sequence (5' to 3') | Usage | Source |
|---|---|---|---|
| UF_rggC_JIM | AAAACTGCAAGTAGAGTCGC CGAATTAGAA | Linear DNA fragment amplified from strain JM1175 (Δ*scuR::erm*) and used as donor DNA | (7) |
| DR_rggD_SS | TAGCTTCATTCATGTCATGTG TCGTCAAAA | | (7) |

**EMSAs**

| Name | Sequence (5' to 3') | Usage | Source |
|---|---|---|---|
| Cy3-Fw.ComSboxSth.com. direct | ATAGAAATGGTGGTGACATA AATGTCACTATTTTTTTTAG | Probe Cy3-ComR box of $P_{comS}$ from strain LMD-9 | (1) |
| Rv.ComSboxSth.com.direct | CTAAAAAAAATAGTGACATTT ATGTCACCACCATTTCTAT | | (1) |
