# Supplementary material for: Non-peptide dysbiosis metabolites reprogram a peptide quorum-sensing receptor to induce sustained predation in beneficial streptococci
Source: PLoS Biol. 2026 Mar 13;24(3):e3003718. doi: 10.1371/journal.pbio.3003718 (PMC12998947; doi:10.1371/journal.pbio.3003718)
Supplement: S5 Table — (PDF) [file pbio.3003718.s014.pdf]

S5 Table. Overlapping and cloning PCR sub-fragments

PCR	Primer 1	Primer 2
Erm cassette amplification	lox66-ery	lox71-ery
luxAB-cat amplification	F_luxAB_ATG	R_cat_tRNAthr
Upstream homologous region of $tRNA_{thr}$ locus	UF_tRNAthr	UR_tRNAthr
Downstream homologous region of $tRNA_{thr}$ locus	DF_tRNAthr	DR_tRNAthr
Upstream homologous region of *comR* gene	UFcomRJIM-SS1-4	URcomRJIM-SS1-4
Downstream homologous region of *comR* gene	DFcomRJIM-SS1-4	DRcomRJIM-SS1-4
Diagnostic PCR for *comR* deletion	Up_comR SS1-4	Down_comR_SS1-4
Upstream homologous region of *comS* gene	UF_PcomR_luxAB	UR_comS
Downstream homologous region of *comS* gene	DF_comS	DRcomRJIM-SS1-4
Diagnostic PCR for *comS* deletion	F_comR	Down_comS_SS1-4
Promoter of *comR* for *luxAB* fusion	F_PcomR_tRNAthr	R_PcomR_luxAB_ATG
Promoter of *scuR* for *luxAB* fusion	F_PrggD_tRNAthr	R_PrggD_luxAB_ATG
Promoter of *sarF* for *luxAB* fusion	F_PrggC_tRNAthr	R_PrggC_luxAB_ATG
