# Supplementary material for: Non-peptide dysbiosis metabolites reprogram a peptide quorum-sensing receptor to induce sustained predation in beneficial streptococci
Source: PLoS Biol. 2026 Mar 13;24(3):e3003718. doi: 10.1371/journal.pbio.3003718 (PMC12998947; doi:10.1371/journal.pbio.3003718)

Fig. 2C: raw images of bacteriocin assays

4HPAA 0 mM

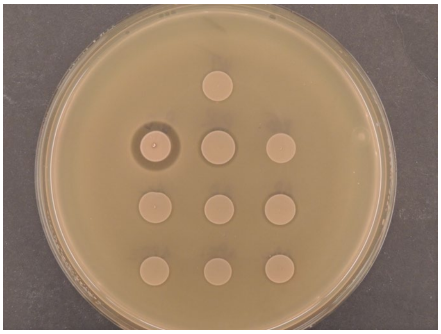

4HPAA 0.1 mM

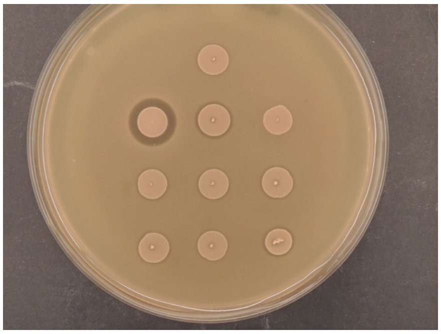

4HPAA 0.25 mM

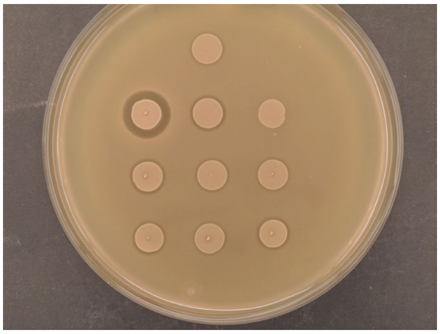

4HPAA 0.5 mM

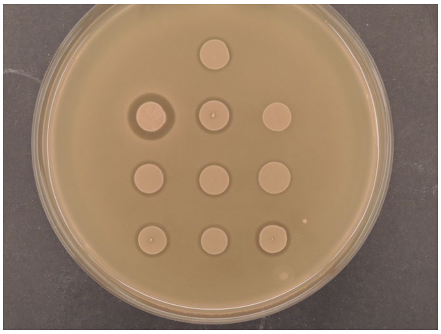

4HPAA 1 mM

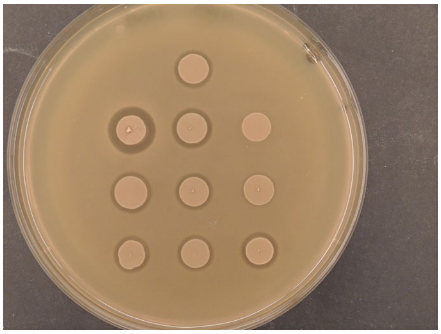

4HPAA 2.5 mM

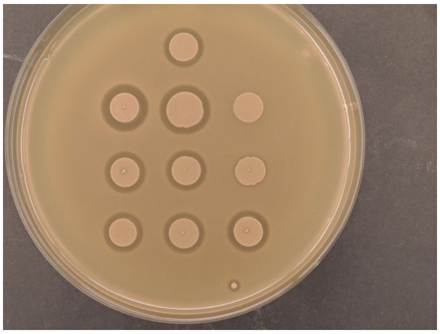

4HPAA 5 mM

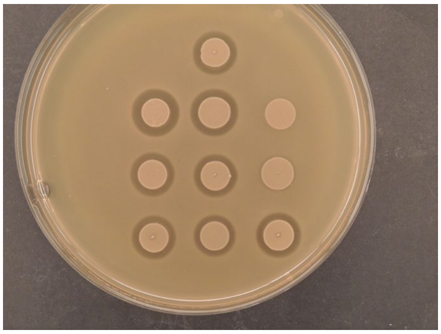

4HPAA 10 mM

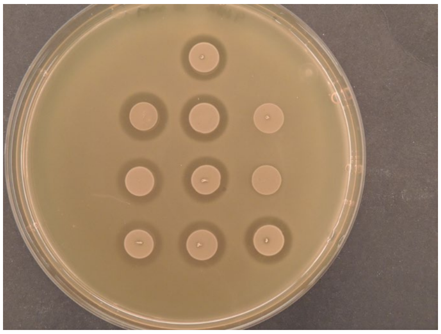

4HPAA 20 mM

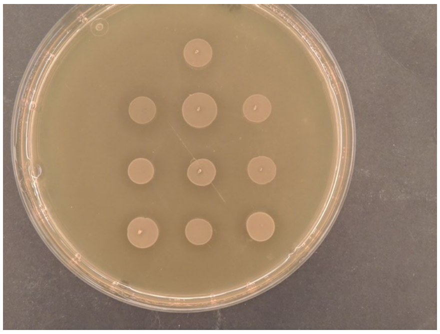

sBI7 1 μM

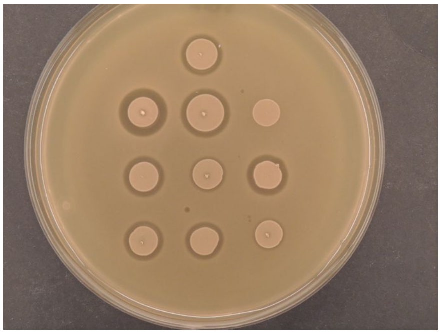

XIP 1 μM

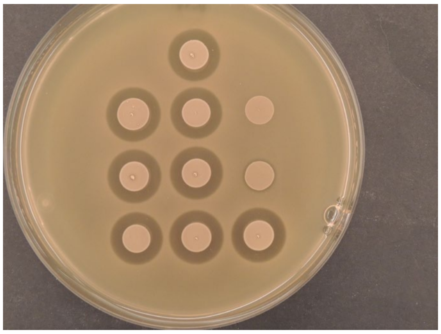

Strain position (mirrored)

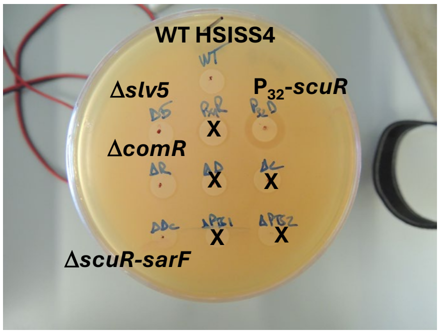

Fig. 2C: raw images of bacteriocin assays

Duplicate test with broader 4HPAAgradient

-

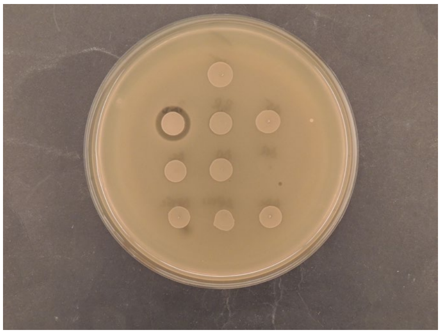

4HPAA 1 µM

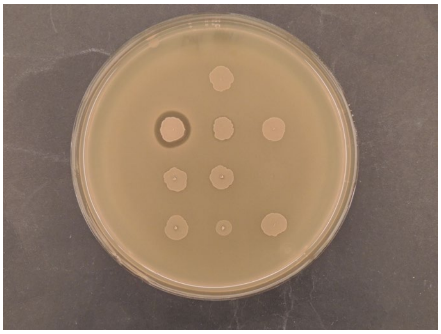

4HPAA 10 µM

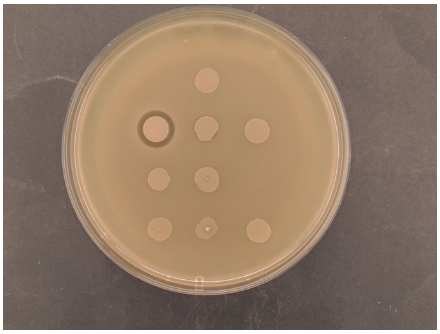

4HPAA 100 µM

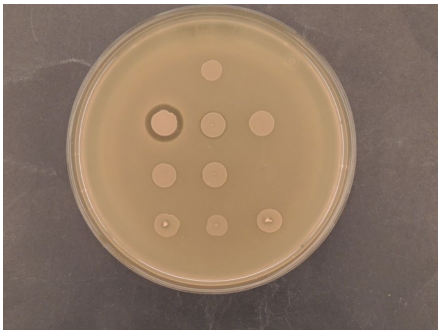

4HPAA 1 mM

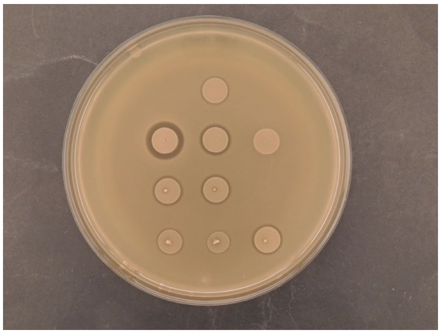

4HPAA 10 mM

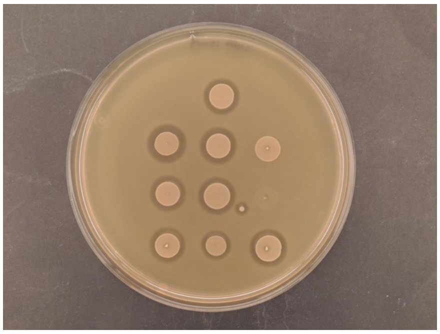

4HPAA 100 mM

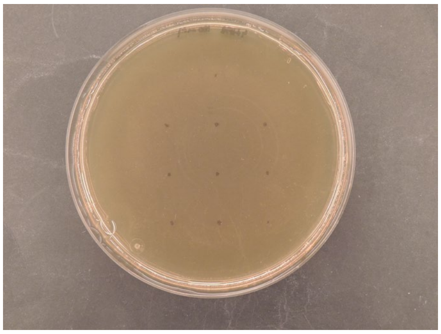

sBI7 1 µM

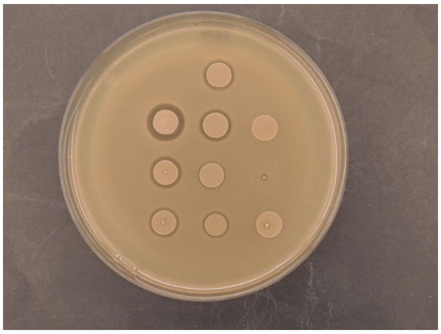

XIP 1 µM

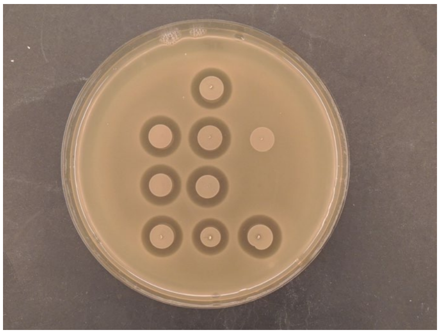

Strain position (mirrored)

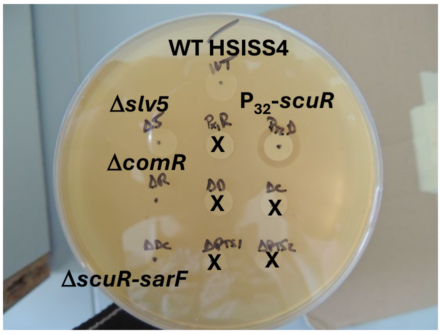

Fig. 2D: raw images of bacteriocin assays (technical replicates)

-

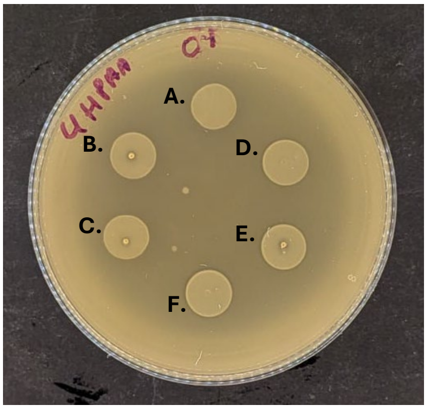

4HPAA 0.1 mM

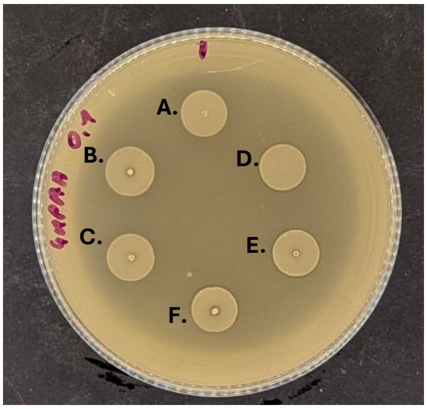

4HPAA 0.25 mM

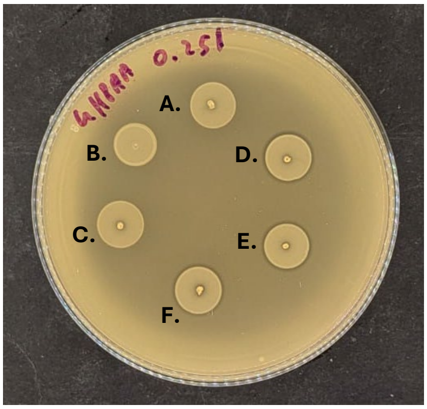

4HPAA 0.5 mM

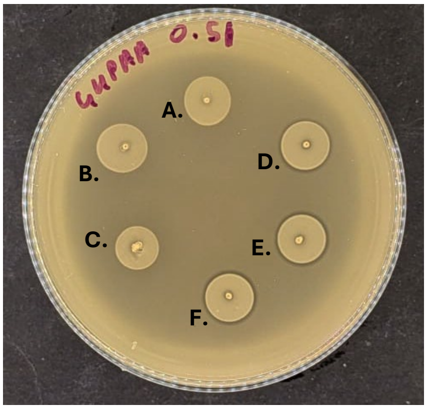

4HPAA 1 mM

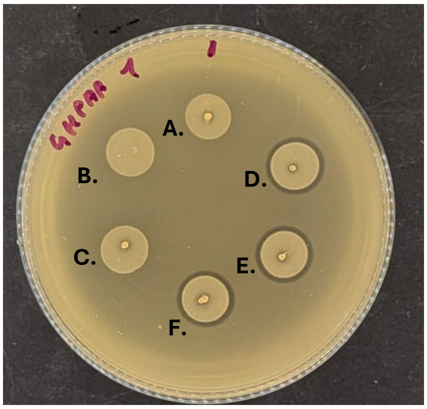

4HPAA 2.5 mM

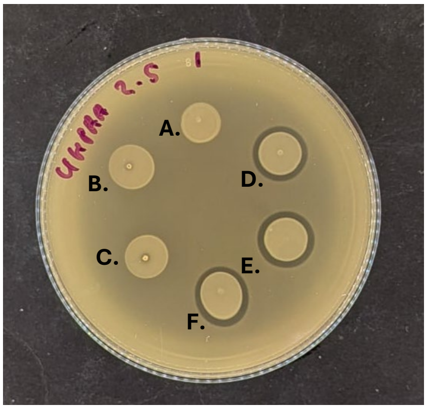

4HPAA 5 mM

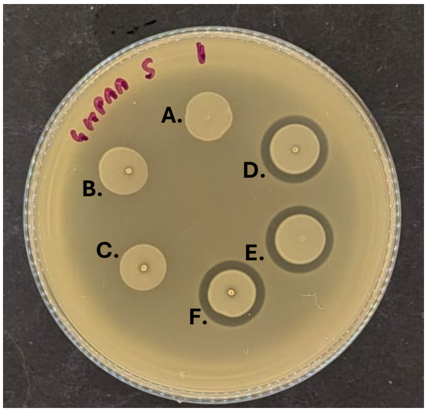

4HPAA 10 mM

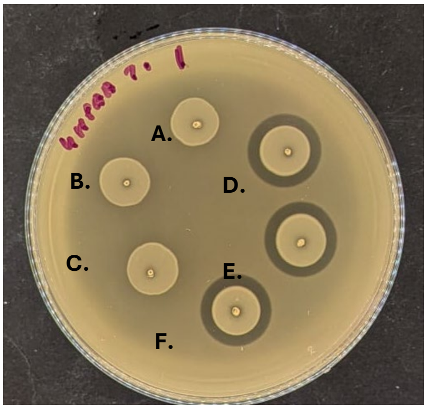

Strain position

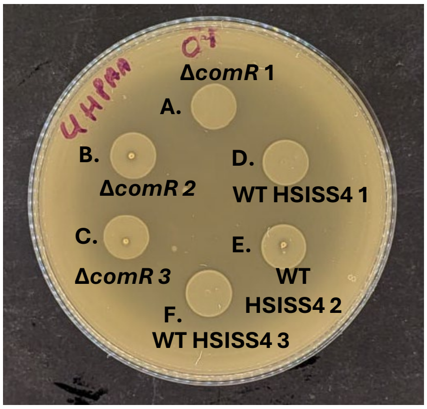

Fig. 3A: raw images of EMSAs

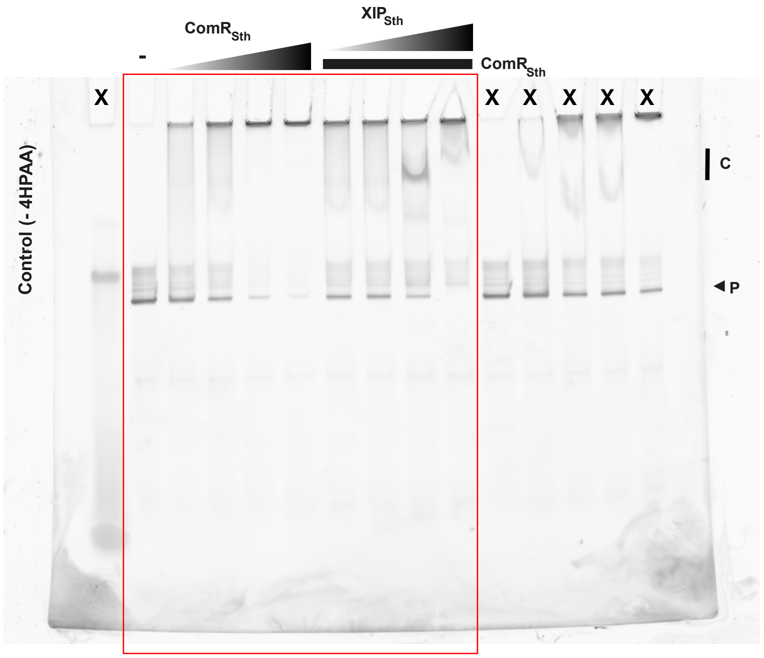

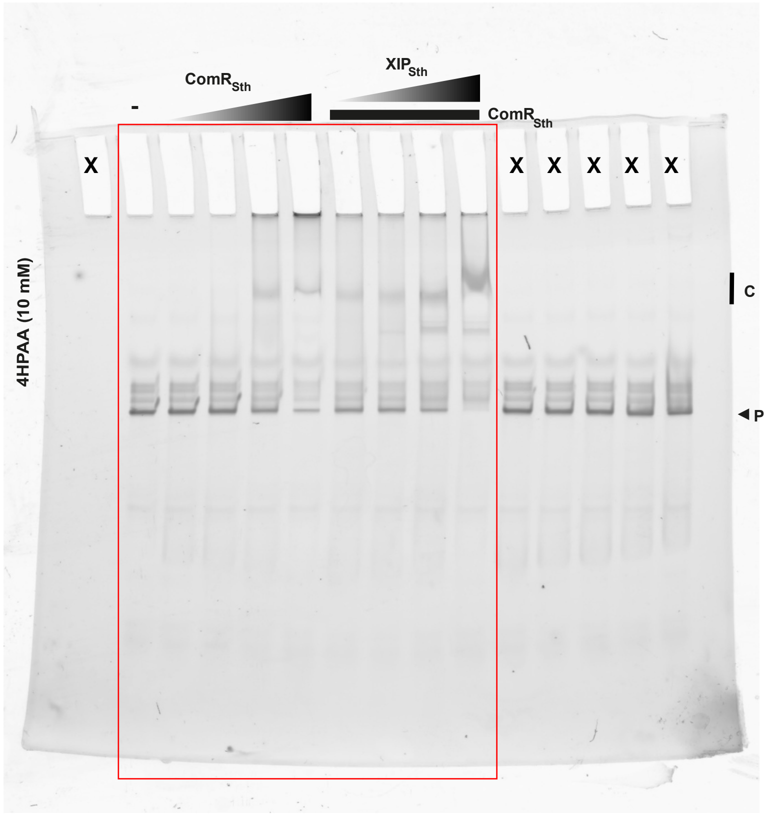

Fig. 5D: raw images of bacteriocin assays (technical replicates)

4HPAA 0 mM + PAA 0 mM

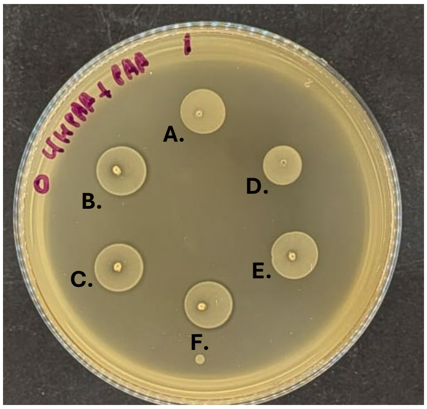

4HPAA 0.25 mM + PAA 0.25 mM

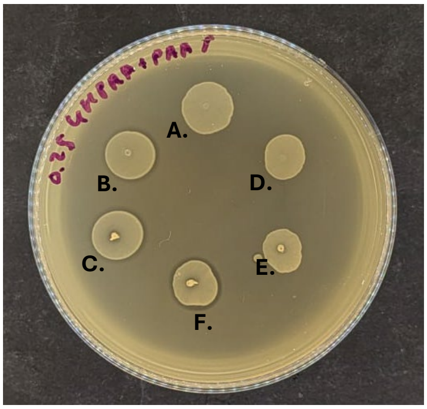

4HPAA 0.5 mM + PAA 0.5 mM

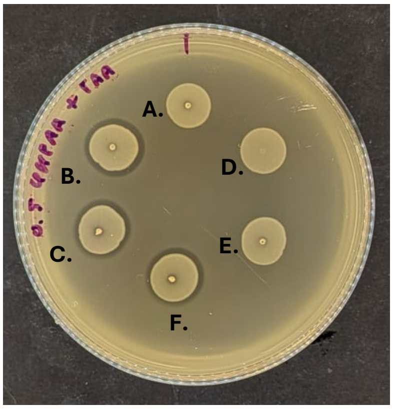

4HPAA 1 mM + PAA 1 mM

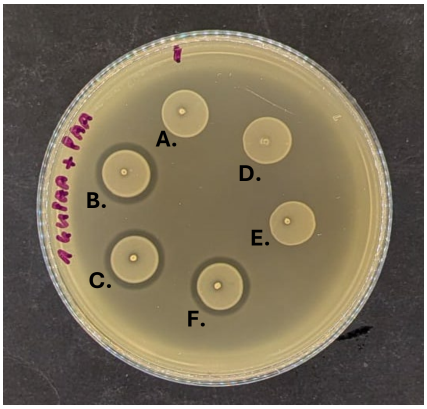

4HPAA 2 mM + PAA 2 mM

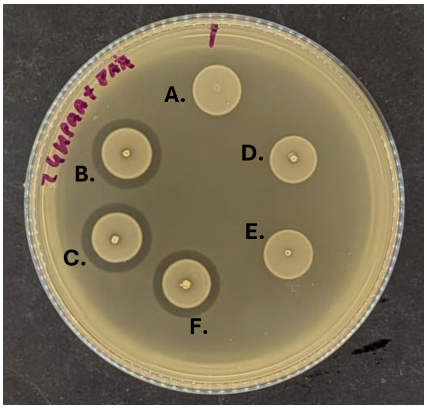

4HPAA 3 mM + PAA 3 mM

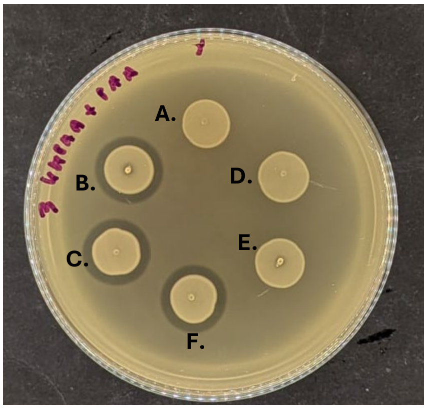

4HPAA 4 mM + PAA 4 mM

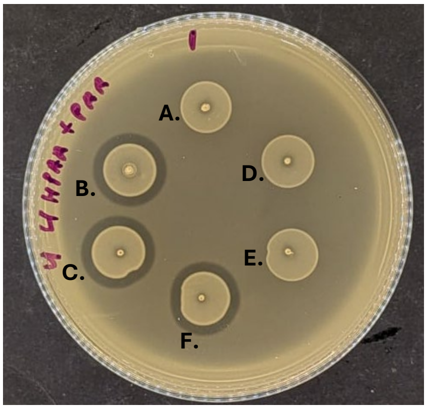

4HPAA 5 mM + PAA 5 mM

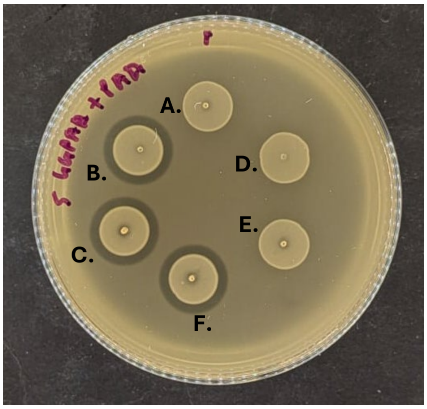

Strain position

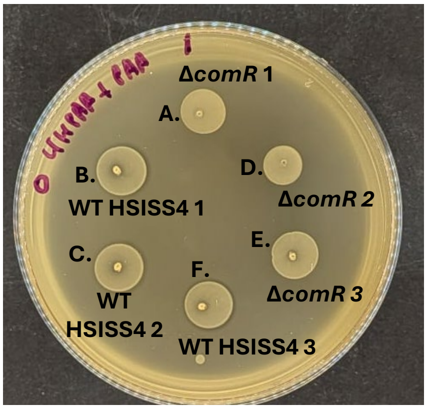

Fig. S3: raw images of EMSAs

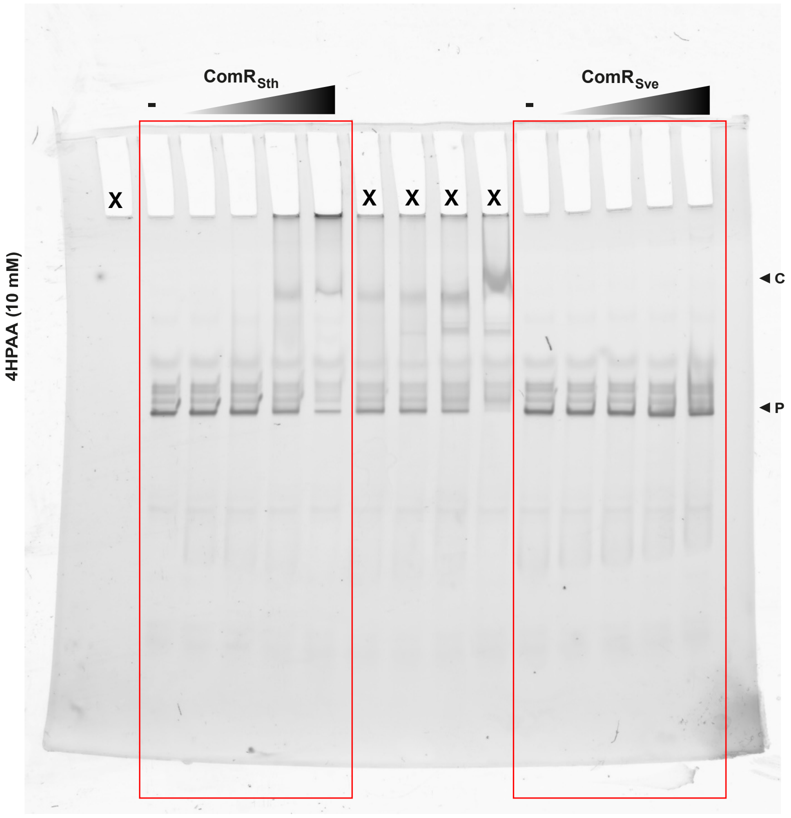

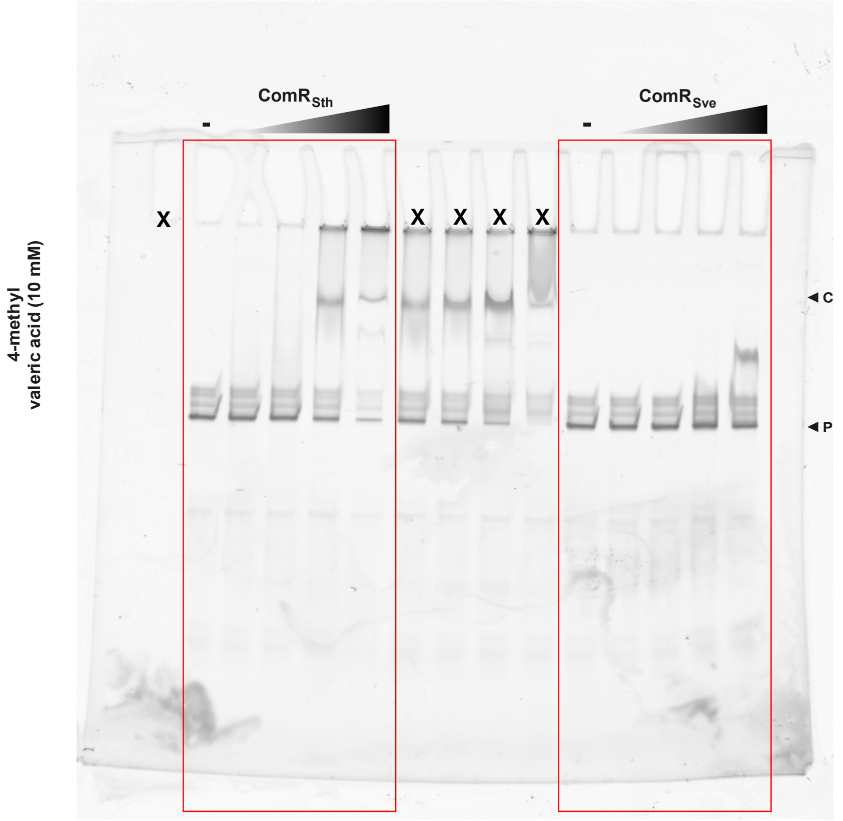

Fig. S8F: raw images of bacteriocin assays

Porphyromonas gingivalis as indicator strain

Replica #1

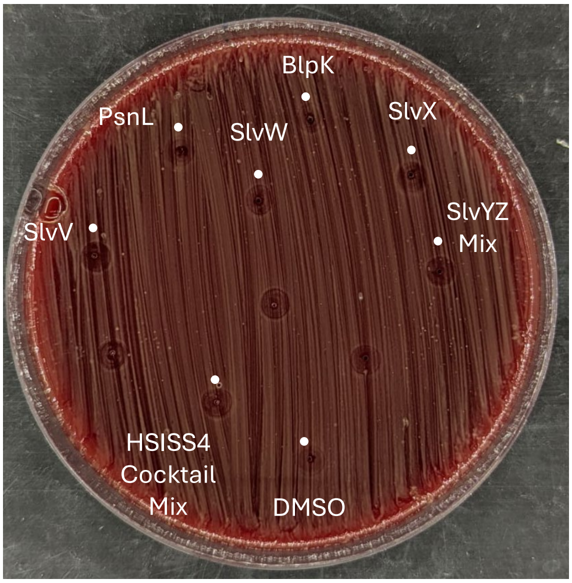

Replica #2

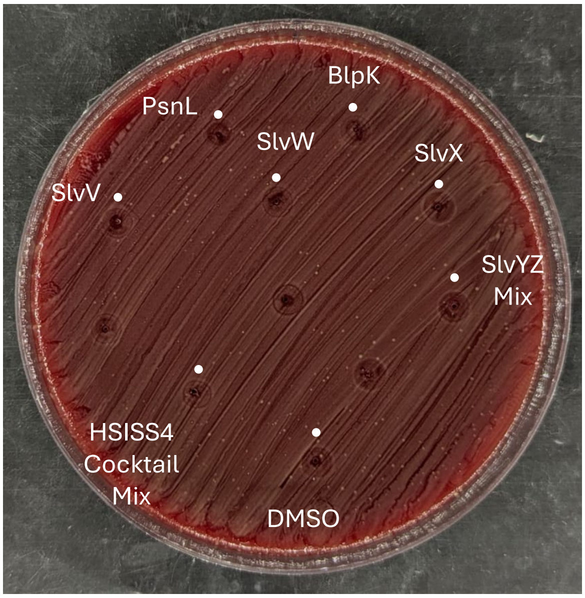

Replica #3

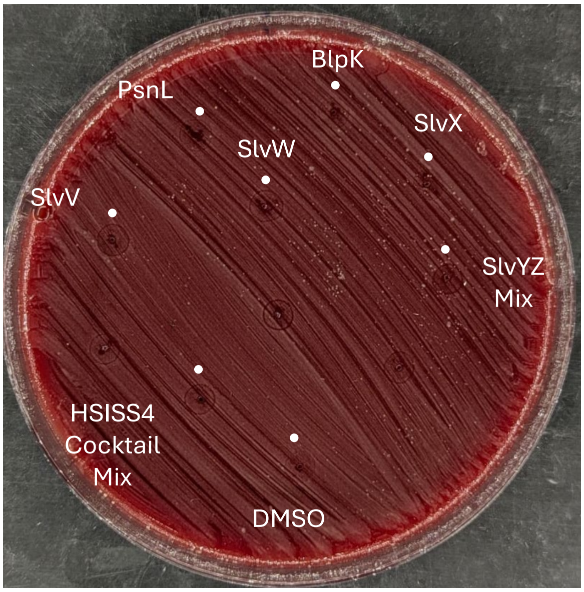

Fig. S8F: raw images of bacteriocin assays

Streptococcus gordonii as indicator strain

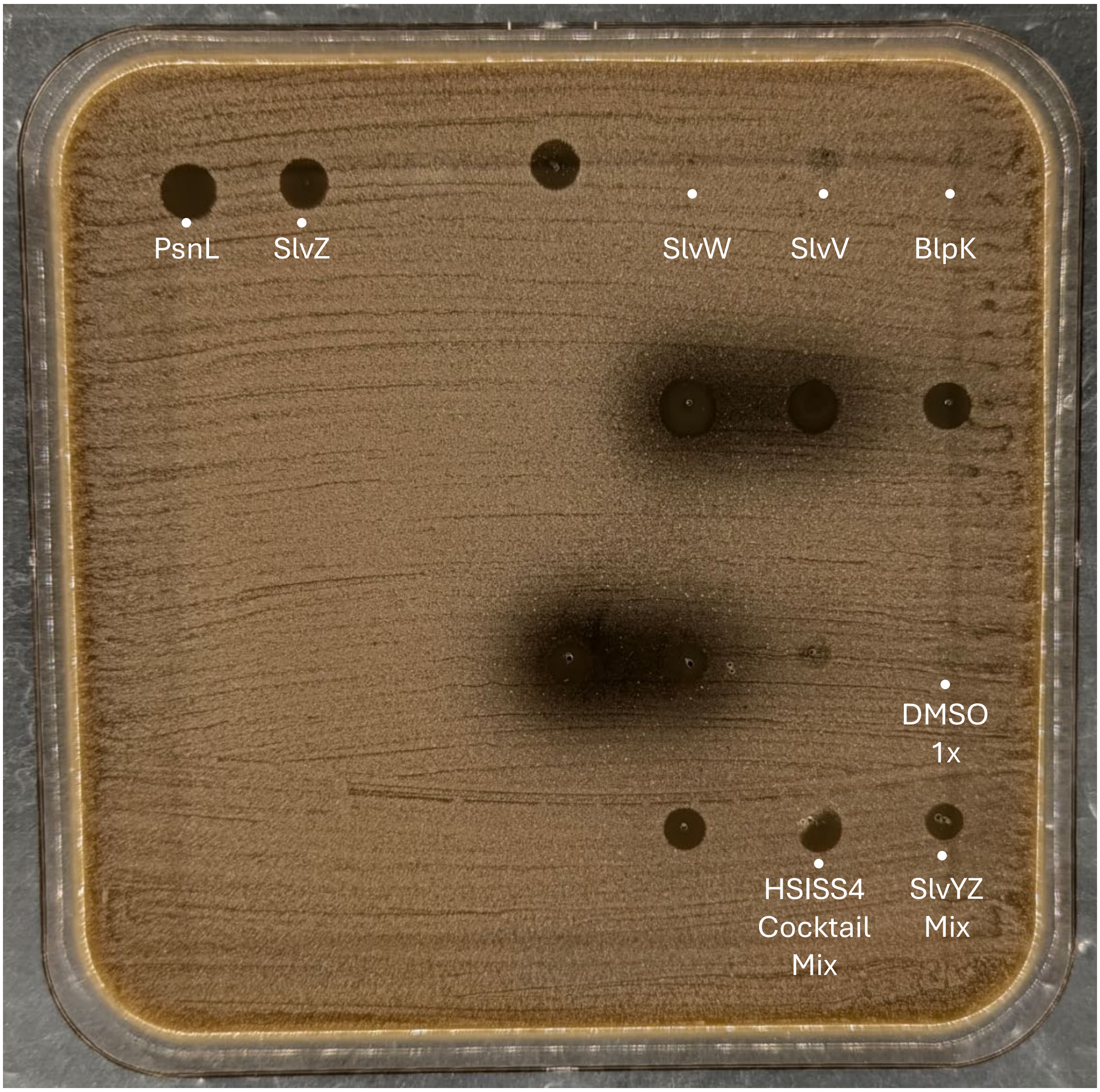

Supplement: S1 Raw Images — (PDF) [file pbio.3003718.s016.pdf]
